# PIM2‐mediated phosphorylation contributes to granulosa cell survival via resisting apoptosis during folliculogenesis

**DOI:** 10.1002/ctm2.359

**Published:** 2021-03-09

**Authors:** Lei Wang, Yaru Chen, Shang Wu, Ling Wang, Feng Tan, Fenge Li

**Affiliations:** ^1^ Key Laboratory of Swine Genetics and Breeding of Ministry of Agriculture and Rural Affairs & Key Laboratory of Agricultural Animal Genetics Breeding and Reproduction of Ministry of Education Huazhong Agricultural University Wuhan China; ^2^ The Cooperative Innovation Center for Sustainable Pig Production Wuhan China

Dear Editor,

Premature ovarian insufficiency (POI) is one of the most common reproductive endocrine disorders, which reduces female fertility.^1^ Dysregulated apoptosis of granulosa cells (GCs) is a major cause of POI with follicular development retardation among young women.^2^ Proviral integration site 2 (PIM2) as a pro‐survival kinase plays a crucial role in repressing apoptotic process in various cell types.^3^ Herein, our study revealed a crucial role of PIM2 in maintaining GC survival during folliculogenesis, discovered a novel antiapoptotic mechanism of PIM2–DAPK3 (death‐associated protein kinase 3)–p53 axis, and provided new targets for POI therapy.

The PIM2 kinase‐inhibited mice generated using C57BL/6J mice treated with SMI‐16a (a PIM2 kinase inhibitor) showed smaller ovaries with the reduced ratio of ovarian weight to body weight, compared with control mice (Figures 1A and 1B). Furthermore, PIM2 kinase‐inhibited mice had a series of phenotypes analogous to POI in women including a decreased serum estradiol level and elevated FSH and LH levels (Figures 1C–1E). Moreover, compared with control group, PIM2 kinase inhibition in mice caused impaired ovarian follicle development with evidences of nearly 50% reduction of primordial follicles and the retardation of developmental transition from secondary follicles to antral follicles (Figures 1F and 1G), which suggests that PIM2 kinase inhibition can impair the primordial follicle reserve and the development of secondary follicles. To decipher the cause of follicle development retardation by PIM2 kinase inhibition, cell proliferation and apoptosis were analyzed by Ki67 and TUNEL staining. Compared with control ovaries, PIM2 kinase inhibited ovaries contained fewer Ki67‐positive and more TUNEL‐positive GCs (Figures 1H–1K). These findings provide evidences to verify the role of PIM2 in follicle development and ovarian reserve.

**FIGURE 1:**
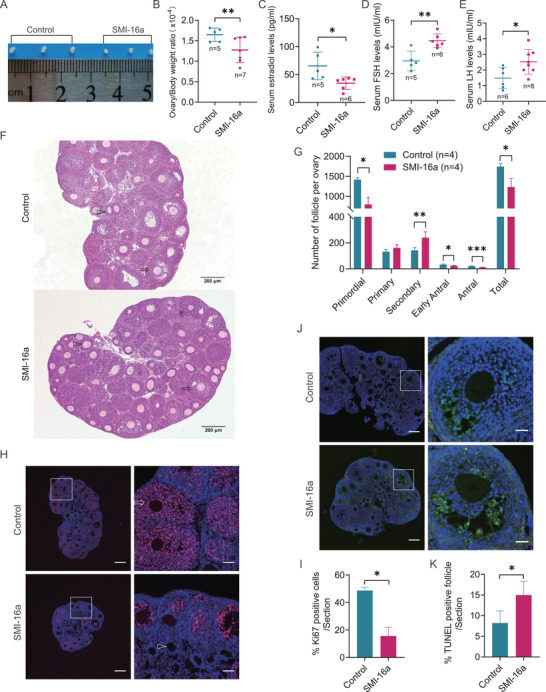
PIM2 kinase inhibition causes the deficiency of follicle development in mice. (A) The representative ovarian morphology. (B) The ratio of ovarian weight to body weight. (C–E) Assessment of serum hormone levels in PIM2 kinase‐inhibited and control mice. (F) Staining of ovarian sections of 1‐month‐old PIM2 kinase‐inhibited mice and control mice. The arrows point to antral follicles and the triangles indicate secondary follicles. Scale bars: 200 μm. (G) The quantification of the number of follicles at different stages of development. (H) Immunofluorescence staining cell proliferation marker Ki67 (red) in PIM2 kinase‐inhibited and control ovaries. The left panels showed the representative micrographs, with the amplified views of the white box area on the right side. Scale bars: 100 and 200 μm. (I) The quantification of the Ki67‐positive cells in (H). (J) TUNEL staining (green) of granulosa cell apoptosis in the ovarian follicles. The left panels showed the representative micrographs, with the amplified views of the white box area on the right side. Scale bars: 50 and 200 μm. (K) The quantification of the TUNEL‐positive cells in panel J. DNA was counterstained with DAPI. *P < 0.05 and **P < 0.01 compared with controls according to two‐tailed Student's *t* test

Subsequently, the role of PIM2 in regulating GC apoptosis and ovarian follicle development in vitro was investigated. We found that *Pim2* knockdown resulted in an increase of apoptosis signal and a decrease of proliferating cells in GCs (Figures 2A‐‐2D). In addition, *Pim2* knockdown inhibited the expression of steroidogenic and ovarian follicle development genes,^4^, ^5^ that is, *Cyp19a1*, *Hsd17b1*, and *Lhr* (Figures 2E and 2F). As CYP19A1 is a key rate‐limiting aromatase enzyme for the estradiol synthesis,^6^ we next measured the effect of PIM2 on estradiol synthesis and found that *Pim2* knockdown could suppress estradiol synthesis (Figure 2G). Likewise, these results were further verified in GCs overexpressing *Pim2* (Figures 2H–2K and S1A–S1C). Furthermore, the kinase‐dead PIM2 mutant (PIM2^K61A^)^7^ could abolish PIM2‐mediated suppression of apoptosis and promotion of cell proliferation (Figures 2H–2K and S1D–S1F). These results suggest that PIM2 promotes GC growth in a kinase‐dependent manner.

**FIGURE 2:**
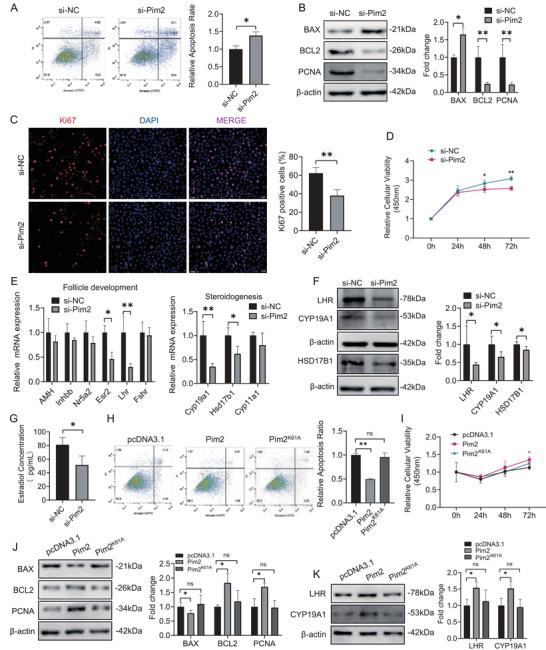
PIM2 is required for granulosa cell (GC) survival in a kinase‐dependent way. (A) Assessment of apoptosis using Annexin V‐FITC/PI and flow cytometry. *Pim2* knockdown was established by employing small interfering RNAs (siRNAs). GCs were transfected with the siRNA‐NC or siRNA‐*Pim2* for 48 h and assayed by flow cytometry. The quantification of apoptotic cells based on the apoptosis assessment was shown in the bar graph. siRNA‐NC is abbreviated to si‐NC. siRNA‐*Pim2* is abbreviated to si‐Pim2. (B) Western blot analysis of BAX, BCL2, and PCNA levels in GCs. GCs were transfected with siRNA‐NC and siRNA‐*Pim2* for 48 h and cell lysates were collected for assays. β‐Actin was used as an internal control. The quantification of protein level was shown in the bar graph. (C) Immunofluorescence staining of cell proliferation marker Ki67 in *Pim2* knockdown (*Pim2*‐KD) and control GCs. Anti‐Ki67 and CY3‐conjugated goat antirabbit IgG (H+L) antibodies were used to detect Ki67 (red). The nuclei were stained by DAPI (blue). The right graph is the quantification of Ki67‐positive cells. Scale bar: 50 μm. (D) Cell viability analysis using CCK‐8 assay in ovarian GCs. GCs were transfected with siRNA‐NC and siRNA‐*Pim2* and then used for detection at 0, 24, 48, and 72 h. (E) Quantitative RT‐PCR analysis of *Amh*, *Inhbb*, *Nr5a2*, *Esr2*, *Lhr*, *Fshr, Cyp19a1*, *Hsd17b1*, and *Cyp11a1* expression in *Pim2*‐KD and control GCs. The expression level of each transcript in controls was set as 1. (F) Western blot analysis of LHR, CYP19A1, and HSD17B1 protein levels in *Pim2*‐KD GCs. β‐Actin was used as an internal control. (G) The quantitative measurement of estradiol concentrations. The siRNA‐NC and siRNA‐*Pim2* were transfected into GCs for 48 h and the media were collected and used for assay. (H) The apoptosis assessment using Annexin V‐FITC/PI and flow cytometry. GCs were transfected with pcDNA3.1 or pcDNA3.1‐*Pim2* or pcDNA3.1‐*Pim2^K61A^*. The right bar graph showed the quantification of apoptosis rate. pcDNA3.1‐*Pim2* and pcDNA3.1‐*Pim2^K61A^* is abbreviated to Pim2 and Pim2^K61A^, respectively. (I) Cell viability analysis of ovarian GCs overexpressing *Pim2* or *Pim2^K61A^* using CCK‐8 assay. (J–K) Western blot analysis of BAX, PCNA, PCNA, LHR, and CYP19A1 expression in GCs overexpressing *Pim2* or *Pim2^K61A^*. β‐Actin was used as an internal control. The right bar graph showed the quantification of protein level. The quantification of protein level was shown in the bar graph. Data were presented as mean ± SEM. *P < 0.05 and **P < 0.01

To identify the downstream phosphoproteins and novel PIM2 substrates, the global quantitative phosphoproteomic analysis in *Pim2* knockdown (*Pim2*‐KD) and control GCs was performed in our workflow and successfully verified by western blot (Figures 3A and S2A). In total, we identified 81 proteins comprising 87 phosphosites with increased phosphorylation levels (fold change > 1.3, *P* < 0.05) and 260 proteins comprising 425 phosphosites with decreased phosphorylation levels (fold change < 0.77, *P* < 0.05) in *Pim2*‐KD GCs, compared with control GCs (Figures S2B and S2C; Table S1). Functional enrichment analysis of differentially regulated phosphoproteins suggested that PIM2 is an important regulator of apoptotic process (GO: 0006915) and cell cycle (GO: 0007049) (Figure 3B). A subset of downregulated phosphoproteins was enriched in term of “positive regulation of apoptotic process” (GO: 0043065), which might provide potential apoptotic substrates of PIM2 (Figure S2D). Notably, some upregulated phosphoproteins might participate in the steroid biosynthetic process (GO: 0006694) and steroid metabolic process (GO: 0008202), in accord with dysregulation of steroidogenesis in *Pim2*‐deficient GCs (Figure S2E).

**FIGURE 3:**
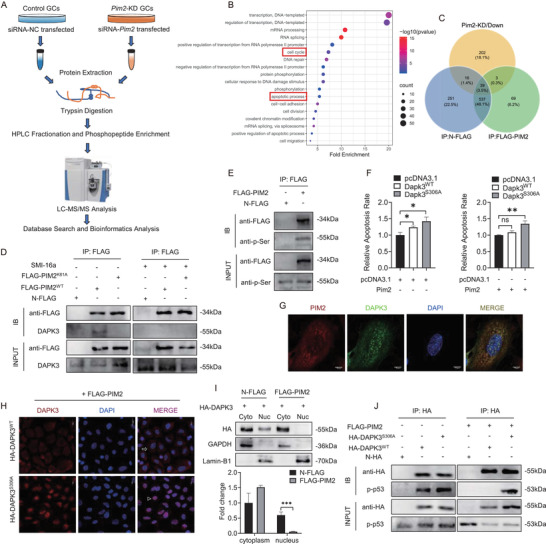
PIM2‐dependent phosphorylation of DAPK3 is necessary for granulosa cell survival. (A) The workflow for the phosphoproteomics experiments. (B) Scatter plot showed the top 18 GO biological process terms of differentially regulated phosphoproteins analyzing using online tools DAVID. Fold enrichment was the ratio of the targeted gene number to the total gene number in a certain term. –Log10 (pvalue) was corrected *P*‐value ranging from 1 to 16. The color and size of the dots represented the range of the –Log10 (pvalue) and the number of phosphoproteins mapped to the indicated terms, respectively. (C) Venn diagram illustrating the relationship of downregulated phosphoproteins in *Pim2*‐KD granulosa cells (GCs) and PIM2 immunoprecipitates. GCs were transfected with N‐FLAG or FLAG‐PIM2 and then cell lysates were immunoprecipitated with anti‐FLAG beads. Immunoprecipitates of PIM2 were identified except that of N‐FLAG by mass spectrum analysis. (D and E) Validation of DAPK3 as a novel PIM2 substrate using immunoprecipitation. Constructs encoding FLAG‐tagged PIM2 were transfected into GCs. The pCMV‐N‐FLAG constructs encoding FLAG tag protein (N‐FLAG) were used for the control of FLAG‐PIM2. Twenty‐four hours after transfection, cells were treated with PIM2 kinase inhibitor SMI‐16a (2 μM) for 24 h (right panel). Cell lysates were subjected to immunoprecipitation assay using anti‐FLAG beads. Western blot was conducted using anti‐FLAG (D) or anti‐p‐Ser antibodies (E). (F) The quantification of apoptosis rate in GCs transfected with indicated plasmids. The apoptosis assessment was measured using Annexin V‐FITC/PI and flow cytometry. (G) Colocalization analysis of endogenous PIM2 with DAPK3 by immunofluorescence assay. Anti‐PIM2 and CY3‐conjugated goat antirabbit IgG (H+L) antibodies were used to detect PIM2 (red). Anti‐DAPK3 and FITC‐conjugated goat antirabbit IgG (H+L) antibodies were used to detect DAPK3 (green). The nuclei were stained by DAPI (blue). Scale bar: 20 μm. (H) Subcellular localization of DAPK3 (red) was confirmed by immunofluorescence assay after *Pim2* overexpression in GCs. White triangles and white arrows indicated representative DAPK3 expression in the nucleus and cytoplasm, respectively. The nuclei were stained by DAPI (blue). Scale bar: 20 μm. (I) Western blot analysis of subcellular localization of DAPK3 protein in GCs. HA‐DAPK3 was co‐expressed with N‐FLAG or FLAG‐PIM2 in GCs. Lamin B1 was used as a nuclear control. GAPDH was used as a cytoplasmic control. The quantification of protein level in nuclei was shown in the bar graph. (J) Immunoprecipitation assay of the interaction between DAPK3 and p‐p53. HA‐tagged DAPK3^WT^ or DAPK3^S306A^ was expressed (left panel) and co‐transfected with FLAG‐PIM2 in GCs (right panel). The pCMV‐N‐HA constructs encoding HA tag protein (N‐HA) were used for the control of HA‐DAPK3. Results are expressed as the mean ± SEM. *P < 0.05 and **P < 0.01

To directly screen out the PIM2 substrates, we immunoprecipitated PIM2 from GCs expressing FLAG‐PIM2 and then identified these immunoprecipitates by mass spectrum (Tables S2 and S3). Subsequently, combination analysis of PIM2 immunoprecipitates and downregulated phosphoproteins in *Pim2*‐KD GCs revealed three potential substrates of PIM2 kinase including DAPK3, RALY (RNA‐binding protein Raly), and LMNB1 (Lamin‐B1) (Figures 3C and S2F). DAPK3, as an apoptotic regulator,^8^ was one of the downregulated phosphoproteins associated with positive regulation of apoptotic process in *Pim2*‐KD GCs (Figure S2G). As expected, PIM2 could bind to and phosphorylate Dapk3 at S306 and this interaction was disappeared in SMI‐16a‐treated GCs or Pim2^K61A^‐overexpressed GCs (Figures 3D and 3E). Moreover, Pim2 knockdown could increase the rate of apoptotic cells (Figure 2A), which was rescued after Dapk3 knockdown in GCs (Figure S3A). In line with an antiapoptotic role, Pim2 could rescue Dapk3^WT^‐induced apoptosis, but not affect the function of phosphorylation‐null Dapk3^S306A^ (Figures 3F and S3B–S3D). However, PIM2 barely affected DAPK3 protein level but co‐localized with DAPK3 in cytoplasm in vivo, and *Pim2* overexpression promoted nuclear exclusion of DAPK3 with evidence of dominant existence of DAPK3^WT^ in the cytoplasm and DAPK3^S306A^ in the nucleus (Figures 3G–3I and S3E). Previous studies have suggested that DAPK3 binds to some transcription factors to coactivate apoptotic pathway in the nucleus and has direct links to the p53 pathway.^9^ Indeed, DAPK3 was able to bind to and phosphorylate p53 (p‐p53), whereas *Pim2* overexpression could abolish the interaction between DAPK3 and p‐p53 in GCs (Figure 3J). Consistently, *Pim2* knockdown in GCs and PIM2 kinase inhibition in mice substantially increased p‐p53 accumulation (Figures S3F and S3G), which was consistent with quantitative phosphoproteomics analysis (Table S1). Collectively, PIM2‐dependent p‐DAPK3 fails to reside in the nucleus and consequently represses p53 transactivation of apoptotic genes, leading to improvement of apoptotic resistance in GCs.

It has been suggested that transcription factor POU2F1 improves oocyte maturation with elevated expression level in antral follicles and activates the hormone‐induced transcription.^5^, ^10^ Subsequently, we identified POU2F1 could specifically bind to the *Pim2* promoter region (–162 to –148 bp and –127 to –113 bp) (Figures S4A–S4G). Moreover, POU2F1 could activate *Pim2* endogenous expression and improve apoptotic resistance in GCs (Figures S5A–S5F).

In conclusion, we generated a PIM2 kinase‐inhibited mouse model characterized by premature loss of ovarian follicles and the dysregulated hormone level that represents the pathogenesis of POI in women. PIM2 as an antiapoptotic kinase is required for GC survival and follicle development in a phosphorylation‐dependent manner. Once kinase activated, PIM2 phosphorylates DAPK3 to inhibit its nuclear translocation, hence leading to repression of p53 transcriptional activity. We provide a schematic model to illustrate the mechanism of PIM2–DAPK3–p53 axis for suppressing GC apoptosis during folliculogenesis (Figure S6). Therefore, this apoptotic resistant machinery might be a promising target for POI therapy.

## AUTHOR CONTRIBUTIONS

FL and LW designed the experiments. LW performed the experiments. FL and LW wrote the manuscript. LW, YC, SW, and FT contributed reagents/materials/analysis tools. LW, YC, and SW analyzed the data.

## CONFLICT OF INTEREST

The authors declare no conflict of interest.

## AVAILABILITY OF DATA AND MATERIALS

The data that support the findings of this study are available from Fenge Li upon reasonable request.

## ETHICS APPROVAL AND CONSENT TO PARTICIPATE

All animals received humane care according to the criteria outlined in the Guide for the Care and Use of Laboratory Animals. All animal experiments were conducted in accordance with the guidelines of the Animal Care and Ethics Committee of Huazhong Agricultural University.

## Supporting information

Supporting information

Supporting information

Supporting information

Supporting information

Supporting information

## References

[1] Wang X , Zhang X , Dang Y , et al. Long noncoding RNA HCP5 participates in premature ovarian insufficiency by transcriptionally regulating MSH5 and DNA damage repair via YB1. Nucleic Acids Res. 2020;48:4480‐4491. 32112110 10.1093/nar/gkaa127 PMC7192606

[2] Sun Z , Zhang H , Wang X , et al. TMCO1 is essential for ovarian follicle development by regulating ER Ca^2+^ store of granulosa cells. Cell Death Differ. 2018;25:1686‐1701. 29467381 10.1038/s41418-018-0067-x PMC6143536

[3] Nawijn MC , Alendar A , Berns A . For better or for worse: the role of Pim oncogenes in tumorigenesis. Nat Rev Cancer. 2011;11:23‐34. 21150935 10.1038/nrc2986

[4] Guo J , Zhang T , Guo Y , et al. Oocyte stage‐specific effects of MTOR determine granulosa cell fate and oocyte quality in mice. Proc Natl Acad Sci USA. 2018;115:E5326‐E5333. 29784807 10.1073/pnas.1800352115 PMC6003357

[5] Zhang Y , Yan Z , Qin Q , et al. Transcriptome landscape of human folliculogenesis reveals oocyte and granulosa cell interactions. Mol Cell. 2018;72:1021‐1034. 30472193 10.1016/j.molcel.2018.10.029

[6] Massillo C , Dalton G , Porretti J , et al. CTBP1/CYP19A1/estradiol axis together with adipose tissue impacts over prostate cancer growth associated to metabolic syndrome. Int J Cancer. 2019;144:1115‐1127. 30152543 10.1002/ijc.31773

[7] Yan B , Zemskova M , Holder S , et al. The PIM‐2 kinase phosphorylates BAD on serine 112 and reverses BAD‐induced cell death. J Biol Chem. 2003;278:45358‐45367. 12954615 10.1074/jbc.M307933200

[8] Farag AK , Roh EJ . Death‐associated protein kinase (DAPK) family modulators: current and future therapeutic outcomes. Med Res Rev. 2019;39:349‐385. 29949198 10.1002/med.21518

[9] Burch L , Scott M , Pohler E , Meek D , Hupp T . Phage‐peptide display identifies the interferon‐responsive, death‐activated protein kinase family as a novel modifier of MDM2 and p21WAF1. J Mol Biol. 2004;337:115‐128. 15001356 10.1016/j.jmb.2003.10.081

[10] Belikov S , Astrand C , Holmqvist P , Wrange O . Chromatin‐mediated restriction of nuclear factor 1/CTF binding in a repressed and hormone‐activated promoter in vivo. Mol Cell Biol. 2004;24:3036‐3047. 15024090 10.1128/MCB.24.7.3036-3047.2004 PMC371135

