# Supplementary material for: PIM2‐mediated phosphorylation contributes to granulosa cell survival via resisting apoptosis during folliculogenesis
Source: Clin Transl Med. 2021 Mar 9;11(3):e359. doi: 10.1002/ctm2.359 (PMC7943893; doi:10.1002/ctm2.359)
Supplement: Supplementary file 1 — Supporting information [file CTM2-11-e359-s001.docx]

**PIM2-mediated phosphorylation contributes to granulosa cell survival via resisting apoptosis during folliculogenesis**

**Running title：**PIM2 kinase promotes granulosa cell survival

**Lei Wang^1^, Yaru Chen^1^, Shang Wu^1^, Ling Wang^1^, Feng Tan^1^, Fenge Li ^1,2*^**

^1^ Key Laboratory of Swine Genetics and Breeding of Ministry of Agriculture and Rural Affairs & Key Laboratory of Agricultural Animal Genetics, Breeding and Reproduction of Ministry of Education, Huazhong Agricultural University, Wuhan 430070, PR China

^2^ The Cooperative Innovation Center for Sustainable Pig Production, Wuhan 430070, PR China

***Corresponding author:** Fenge Li, Key Laboratory of Pig Genetics and Breeding of Ministry of Agriculture and Rural Affairs & Key Laboratory of Agricultural Animal Genetics, Breeding and Reproduction of Ministry of Education, Huazhong Agricultural University; The Cooperative Innovation Centre for Sustainable Pig Production, Wuhan 430070, PR China. Tel: 86-27-87282091; Fax: 86-27-87280408; E-mail: [lifener@mail.hzau.edu.cn](mailto:lifener@mail.hzau.edu.cn)

**Methods**

**Animals and collection of ovaries**

All mice were housed in a pathogen-free environment with the temperature maintained at 20-22°C and relative humidity at 50-70%, and were under a 12 h/12 h light/dark cycle. All mice had *ad libitum* accesses to standard chow diet. Female Kunming (3-4 weeks of age) mice purchased from the Center for Disease Control (CDC; Hubei, China) were injected intraperitoneally (i.p.) with 10 unit of pregnant mare serum gonadotropin (PMSG; Ningbo Second Hormone Factory).^1^ After 48 h, mouse ovaries were harvested for in *vitro* experiments. Female C57BL/6J mice (postnatal 10 days) were intraperitoneally injected with PIM2 inhibitor, SMI-16a (S6497, Selleck) at 50 mg/kg to generate PIM2 kinase inhibited mice or coil oil to generate control mice every other day.^2^

**Cell culture and cell transfection**

Granulosa cell from pre-ovulatory ovarian follicles of Kunming mouse was isolated as described previously.^1^ CHO-K1 cells (GDC018) were purchased from the China Centre for Type Culture Collection (Shanghai, China). Murine primary granulosa cells and CHO-K1 cells were cultured in Dulbecco’s minimum essential medium/nutrient (DMEM)/F-12 (11320033, Gibco) or (SH30256.01, HyClone) supplemented with 10% fetal bovine serum (10099141C, Gibco), 100 unit/mL penicillin and 100 mg/mL streptomycin (15140122, Gibco) at 37 °C in a humidified atmosphere of 5% CO_2_. Cells were inoculated in different sizes of plates and grew up to 70% confluent at the time of transfection. Plasmids and siRNAs were transfected into the cells using Lipofectamine™ 3000 (L3000015, Invitrogen™) and RNAiMAX transfection reagent (13778030, Invitrogen™), respectively.^3^

**RNA interference and plasmids construction**

The synthesized siRNA-*Pim2* (si-*Pim2*), siRNA-*Pou2f1* (si-*Pou2f1*) or siRNA-NC (si-NC) was purchased from GenePharma (Suzhou, Jiangsu, China). The sequences of the siRNAs are shown in Table 1.

cDNAs with PIM2 (NM_138606.2) shortest isoform were cloned into pcDNA3.1 and pCMV-N-FLAG to generate pcDNA3.1-*Pim2* and FLAG-PIM2. Full-length *Dapk3* (NM_001190473.1) was cloned into pcDNA3.1 and pCMV-N-HA to generate pcDNA3.1-*Dapk3* and HA-DAPK3, and full-length *Pou2f1* (NM_001368808.1) was cloned into pcDNA3.1 to generate pcDNA3.1-*Pou2f1.* Site-directed mutagenesis for the DAPK3 phosphosite and POU2F1 binding site was performed using the primers described in Table 1. Truncated fragments of the mouse *Pim2* promoter were amplified from mouse genomic DNAs and cloned into the pGL3-basic vector.

**Luciferase reporter assay**

Each plasmid was transfected with 500 ng recombinant constructs, together with 50 ng/well of pRL-TK (E2241, Promega). After transfection for 24 h, cells were collected and luciferase activities were measured using the Dual-Luciferase Reporter Assay System (E1910, Promega) according to the manufacturer’s instructions. The experiments were repeated at least 3 times, and the results were expressed as the means ± SD.

**Quantitative real-time PCR**

Total RNA was using the TRIzol Reagent (15596026, Invitrogen™) following the manufacturer’s instructions. Then total RNA was treated with RNase-free DNase (M610A, Promega). Synthesis of cDNA was performed using 1 μg total RNA from each sample using RevertAid RT Reverse Transcription Kit (K1691, Thermo Scientific™). The reverse transcription reaction products were directly used in PCR. Quantitative real-time PCR was carried on by the iTaq^TM^ Universal SYBR Green Super Mix (172-5121, Bio-Rad) and analyzed on CFX384 Touch™ Real-Time PCR Detection System (Bio-Rad, USA). Fold changes of interested genes were computed using the 2^−ΔΔCt^ method. The primers and PCR conditions are listed in Table 1.

**Western blot**

Western blot analysis was performed following the standard procedures.^4^ Briefly, protein lysates were generated using RIPA Lysis Buffer (Beyotime, P0013B) with 1% Protease inhibitor cocktail (HY-K0010T, MCE) and 1% Phosphatase Inhibitor Cocktail I (HY-K0021, MCE) and 1% Phosphatase Inhibitor Cocktail II (HY-K0022, MCE). Protein extracts (30 μg) were separated in 10% SDS-polyacrylamide gels and then transferred onto 0.2 μm PVDF membranes (iseq00010, Millipore). The membranes were then blocked with 5% non-fat dried milk or 3% BSA (Bull Serum Albumin) in TBST (20 mmol/L Tris-HCl, pH 7.5, 150 mmol/L NaCl, 0.1% Tween-20) and then incubated with specific primary antibodies following horseradish peroxidase-conjugated secondary antibodies. The ECL kit (170-5061, Bio-Rad) was used to detect immunoreactive protein bands by ChemiDocMPImaging System (Bio-Rad, USA). ImageJ was used to quantify the signal. The details of the antibodies are listed in Table 2.

**Immunoprecipitation**

Cell lysates were incubated with immunomagnetic beads with anti-FLAG tag antibody (B26101, Bimake) or anti-HA tag antibody (B26201, Bimake) overnight at 4°C. The beads were collected by centrifugation and then washed with PBST buffer (0.1% Tween-20 in PBS). Bound proteins were eluted with loading buffer (50 mM Tris-HCl, 2% SDS, 1% mercaptoethanol, 10% glycerol, 0.1% bromophenol blue, pH 6.8), separated by SDS-PAGE and immunoblotted with appropriate antibodies according to standard procedures.

**Chromatin immunoprecipitation**

The ChIP experiment was carried on using the EZ-Magna ChIP™ A/G Kit (17-371, Millipore). GCs were fixed in 1% formaldehyde and quenched by 0.125 M glycine. Then the isolated chromatin DNA was sonicated and sheared to a length between 200 bp and 800 bp by the AVCX130 system (Sonics & Materials, Newtown, CT, USA). The sheared chromatin was immunoprecipitated with anti-HA or a homologous IgG used as the negative control. The co-precipitated DNA was purified by DNA Purification Kit (D0033, Beyotime) and then subjected to qPCR. The details of antibodies and specific primers for ChIP-qPCR are listed in Table 1, respectively.

**Fluorescence-activated cell sorting (FACS)**

The apoptosis rate of GCs was measured using an Annexin V-FITC Apoptosis Detection Kit (AD10, Dojindo). Briefly, GCs were harvested through trypsinization without EDTA (15050065, Gibco™) and washed with PBS. Following centrifugation at 1000 rpm for 5 min, the sediment was re-suspended in 500 μL Binding Buffer and incubated with 5 μL FITC-conjugated Annexin V and 5 μL PI for 10 min. The samples were analyzed by FACS Calibur Flow Cytometry (Beckman Coulter, Brea, USA).

**TUNEL staining**

To detect cellular apoptosis in the ovaries, 4% PFA fixed ovarian paraffin sections were prepared. TUNEL staining was then performed using the Fluorescein (FITC) Tunel Cell Apoptosis Detection Kit (G1501, Servicebio) according to the manufacturer’s instructions. The sections were fixed with 4% paraformaldehyde for 20 min at room temperature and permeabilized with 20 μg/mL proteinase K (P2308, Sigma-Aldrich) for 10 min at room temperature, and then the sections were incubated with the TdT mix containing FITC-dUTP for 1 h at 37 °C. The nuclei were stained by propidium iodide (PI) for 2 min. Images were captured by an epifluorescence microscope (Olympus BX53).

**Cell viability assay**

Cell viability at 0, 24, 48, and 72 h post-transfection was determined using Cell Counting Kit-8 (CCK-8) (CK04, Dojindo). Briefly, GCs were inoculated in 96-well plates and incubated with 10 mL of CCK-8 assay solution at 37°C in a humid atmosphere containing 5% CO_2_ for 2 h. Living cells promoted the formation of formazan and the absorbance values at 450 nm were measured via the PE Enspire Multimodal Plate Reader (PerkinElmer, USA).

**In silico sequence analysis**

Neural network promoter prediction software (http://www.fruitfly.org/seq_tools/promoter.html) was used to analyze the potential promoters and the TRANSFAC® Public 6.0. (<http://gene-regulation.com/pub/> programs.html) was used to predict the transcription factor binding sites on the *Pim2* promoter region. The mission of Gene Ontology analysis was performed by Gene ontology (<http://geneontology.org/>), David (https://david.ncifcrf.gov/) and Metascape (<http://metascape.org/gp/index.html#/main/step1>). The schematic summary was created with BioRender.com.

**Serum hormone measurement**

GCs transfected with oligonucleotides or plasmids were cultured in serum-free DMEM/F12 containing 10 μg/mL testosterone for 48 h. The media were collected and detected for the quantitative determination of estradiol concentrations. For measuring serum hormone, mice were anesthetized and blood was collected by cardiac puncture. Serum hormone levels were determined by FSH ELISA Kit (KA2330, Novus Biologicals), Luteinizing Hormone ELISA Kit (KA2332, Novus Biologicals) and Estradiol Assay Kit (KGE014, R&D) following instructions, respectively.

**Histology and Immunofluorescence**

These assays were carried out following the same procedure as previously described.^5^ Briefly, ovaries from one-month-old female mice were fixed in Bouin’s fixative, embedded in paraffin, and serially sectioned at 5 μm thickness. The ovarian sections were then stained with hematoxylin and eosin (Sigma-Aldrich, USA), and follicles at various stages of development were counted on every second section based on morphological classification of mouse follicles throughout the whole ovary.^6^ For immunofluorescence, sections or cells were sequentially incubated with primary antibodies and Alexa Fluor 594- or 488-conjugated secondary antibodies as described previously.^7^ Digital images were acquired using an epifluorescence microscope (Olympus BX53) or confocal fluorescence microscopy (Zeiss LSM 800, Germany) with 4-100× objectives. Semi-quantitative analysis of the fluorescence signals was conducted using the NIH Image analysis program ImageJ. The antibodies used are listed in Table 2.

**Phosphopeptide enrichment and MS Analysis**

GC peptides were labeled with isobaric tags for relative or absolute quantitation (iTRAQ) or tandem mass tags (TMT). Phosphopeptides were enriched using titanium dioxide followed by sequential elution from immobilized metal ion affinity chromatography and fractionation by hydrophilic interaction liquid chromatography (TiSH) as previously described.^8^ The peptides were subjected to NSI source followed by MS/MS in Q ExactiveTM plus coupled online to the UPLC followed a described protocol.^9^

**MS Data Interpretation**

The resulting MS/MS data were processed using Maxquant search engine (v.1.5.2.8). Tandem mass spectrum was searched against mouse uniprot database concatenated with reverse decoy database. Trypsin/P was specified as cleavage enzyme allowing up to 4 missing cleavages. The mass tolerance for precursor ions was set as 20 ppm in First search and 5 ppm in Main search, and the mass tolerance for fragment ions was set as 0.02 Da. Carbamidomethyl on Cys was specified as fixed modification and acetylation modification and oxidation on Met were specified as variable modifications. FDR was adjusted to < 1% and the minimum score for modified peptides was set > 40.

**Statistical analysis**

All results are presented as the mean ± S.E.M. Each treatment had three replicates at least. Two-tailed *t-test* was used when two groups were compared. Significant differences were evaluated using an independent-samples *t-test*. *P*<0.05 was considered to be statistically significant.

**References**

1. Shen M, Jiang Y, Guan Z, et al. Protective mechanism of FSH against oxidative damage in mouse ovarian granulosa cells by repressing autophagy. *Autophagy.* 2017; **13**: 1364-1385.

2. Xia Z, Knaak C, Ma J, et al. Synthesis and evaluation of novel inhibitors of Pim-1 and Pim-2 protein kinases. *Journal of medicinal chemistry.* 2009; **52**: 74-86.

3. Zhou J, Lei B, Li H, et al. MicroRNA-144 is regulated by CP2 and decreases COX-2 expression and PGE2 production in mouse ovarian granulosa cells. *Cell death & disease.* 2017; **8**: e2597.

4. Sheng Y, Song Y, Li Z, et al. RAB37 interacts directly with ATG5 and promotes autophagosome formation via regulating ATG5-12-16 complex assembly. *Cell Death Differ.* 2018; **25**: 918-934.

5. Sha Q, Jiang Y, Yu C, et al. CFP1-dependent histone H3K4 trimethylation in murine oocytes facilitates ovarian follicle recruitment and ovulation in a cell-nonautonomous manner. *Cell Mol Life Sci.* 2020; **77**: 2997-3012.

6. Myers M, Britt K, Wreford N, Ebling F, Kerr J. Methods for quantifying follicular numbers within the mouse ovary. *Reproduction.* 2004; **127**: 569-580.

7. Yuan J, Zhang Y, Sheng Y, Fu X, Cheng H, Zhou R. MYBL2 guides autophagy suppressor VDAC2 in the developing ovary to inhibit autophagy through a complex of VDAC2-BECN1-BCL2L1 in mammals. *Autophagy.* 2015; **11**: 1081-1098.

8. Chen Z, Lei C, Wang C, et al. Global phosphoproteomic analysis reveals ARMC10 as an AMPK substrate that regulates mitochondrial dynamics. *Nature communications.* 2019; **10**: 104.

9. Ye X, Pan T, Wang D, et al. viaFoot-and-Mouth Disease Virus Counteracts on Internal Ribosome Entry Site Suppression by G3BP1 and Inhibits G3BP1-Mediated Stress Granule Assembly Post-Translational Mechanisms. *Frontiers in immunology.* 2018; **9**: 1142.

**Table 1. Sequences of primers and oligos (5’-3’).**

| **Primer name** | **Gene name** | | **Gene Bank accession** | **Sequences (5’-3’)** | | **Application** | **Product length (bp)** |
| --- | --- | --- | --- | --- | --- | --- | --- |
| *Pim2*-F | *Pim2* | | NM_138606 | GGTAAGGGAGGCTTTGGC | | qPCR | 133 |
| *Pim2*-R |  |  |  | CAAGTGGGCAGGTGACTGA | |  |  |
| *Cyp19a1*-F | *Cyp19a1* | | NM_001348171 | ATGTTCTTGGAAATGCTGAACCC | | qPCR | 150 |
| *Cyp19a1*-R |  |  |  | AGGACCTGGTATTGAAGACGAG | |  |  |
| *Cyp11a1*-F | *Cyp11a1* | | NM_001346787 | GGCCCAATTTACAGGGAGAAG | | qPCR | 143 |
| *Cyp11a1*-R |  |  |  | CACCAGGGTACTGGCTGAAG | |  |  |
| *Hsd17b1*-F | *Hsd17b1* | | NM_010475 | ACATGAAGAGGCGCCACTCT | | qPCR | 100 |
| *Hsd17b1*-R |  |  |  | GCAAACTTGCTGGCACAGTACA | |  |  |
| *Lhr*-F | *Lhr* | | NM_001364898 | CGCCCGACTATCTCTCACCTA | | qPCR | 150 |
| *Lhr*-R |  |  |  | GACAGATTGAGGAGGTTGTCAAA | |  |  |
| *Inhbb*-F | *Inhbb*- | | NM_008381 | GTGACAAATGGCACCAATGC | | qPCR | 100 |
| *Inhbb*-R |  |  |  | GTGCTCAGAAGGCGCAATTC | |  |  |
| *Nr5a2*-F | *Nr5a2* | | NM_001159769 | GAGCTCTTGATTCTCGATCACATTTAC | | qPCR | 110 |
| *Nr5a2*-R |  |  |  | GTGTGTGAGATGATGGTGGAGTAGTC | |  |  |
| *Esr2*-F | *Esr2* | | NM_010157 | TGTGCTATGGCCAACTTCTG | | qPCR | 184 |
| *Esr2*-R |  |  |  | AGTAACAGGGCTGGCACAAC | |  |  |
| *Fshr*-F | *Fshr*-F | | NM_013523 | TCTCCAACCTACCCAACTTGCA | | qPCR | 100 |
| *Fshr*-R |  |  |  | TATCGGAGACTGGGAAGATTCTG | |  |  |
| *Amh*-F | *Amh*- | | NM_007445 | GCAGTTGCTAGTCCTACATCTGG | | qPCR | 73 |
| *Amh-*R |  |  |  | TGGAGGCTCTTGGAACTTCAGC | |  |  |
| *β-actin*-F | *Actb* | | NM_007393 | GGCACCACACCTTCTACAATG | | qPCR | 133 |
| *β-actin* -R |  |  |  | GGGGTGTTGAAGGTCTCAAAC | |  |  |
| *Pim2*-mut-K61A-F | *Pim2* | | NM_138606 | CAGGTGGCCATCGCAGTAATCTCCCGGAACCGTGTGCTA | | site-directed mutagenesis PCR | 936 |
| *Pim2*-mut-K61A-R |  |  |  | CCGGGAGATTACTGCGATGGCCACCTGACGTCTATCCG | |  |  |
| *Dapk3*-mut-S306A-F | *Dapk3* | | NM_001190473 | TCAAGTCCCACGCGAGCATGCCGCGCAACA | | site-directed mutagenesis PCR | 1347 |
| *Dapk3*-mut-S306A-R |  |  |  | GGCATGCTCGCGTGGGACTTGAGGCTGTACTCG | |  |  |
| *Pim2*-ChIP-qPCR-F | *Pim2* | | NC_000086 | AACGCAGTTGTCGCAAAGGA | | ChIP-qPCR | 248 |
| *Pim2*-ChIP-qPCR-R |  |  |  | CCGCCCACCTGGCTTTGAT | |  |  |
| si-NC oligo |  | |  | UUCUCCGAACGUGUCACGUTT | | RNAi |  |
| si-*Pim2* oligo | *Pim2* | NM_138606 | | | GGAUUCAGCGGGCUCAAUATT | RNAi |  |
| si-*Pou2f1* oligo | *Pou2f1* | | NM_001368808 | CACCAUCUCUCGCUUUGAATT | | RNAi |  |

**Table 2. The information of antibody.**

| **Protein name** | **Catalogue number (Manufacture)** | **Applications (working dilution)** |
| --- | --- | --- |
| PIM2 | A15598 (Ablonal) | WB (1:2000) |
| PIM2 | PA5-81868(Invitrogen) | IF (1:100) |
| BCL2 | ab182858 (Abcam) | WB (1:2000) |
| BAX | A19684 (Abclonal) | WB (1:2000) |
| PCNA | A12427 (Abclonal) | WB (1:2000) |
| Ki67 | A2094 (Abclonal) | IF (1:200) |
| DAPK3 | A15047 (Abclonal) | WB (1:2000), IF (1:200) |
| POU2F1 | 10387-1-AP (Proteintech) | WB (1:1000) |
| p53 | 2524 (Cell signaling) | WB (1:2000) |
| p-p53 | 2528 (Cell signaling) | WB (1:1000) |
| HA | 3724S (Cell signaling) | WB (1:1000), ChIP (1:50), IF (1:200) |
| FLAG | 20543-1-AP (Proteintech) | WB (1:2000) |
| IgG | 2729S (Cell signaling) | ChIP (1:50) |
| 4EBP1 | 9644S (Cell signaling) | WB (1:1000) |
| p-4EBP1 | 9451 (Cell signaling) | WB (1:1000) |
| TSC2 | 4308 (Cell signaling) | WB (1:1000) |
| p-TSC2 | 3615 (Cell signaling) | WB (1:1000) |
| β-actin | AC026 (Abclonal) | WB (1:10000) |
| LAMIN B1 | A11495 (Abclonal) | WB (1:2000) |
| GAPDH | A19056 (Abclonal) | WB (1:2000) |
| CYP19A1 | A2161 (Abclonal) | WB (1:1000) |
| LHR | AF9104 (Affbiotech) | WB (1:1000) |
| HSD17B1 | A10839 (Abclonal) | WB (1:2000) |
| Goat Anti-Rabbit IgG (H + L)-HRP Conjugate | 1706515 (Bio-rad) | WB (1:3000) |
| Goat Anti-Mouse IgG (H + L)-HRP Conjugate | 1706516 (Bio-rad) | WB (1:3000) |
| FITC Goat Anti-Rabbit IgG (H+L) | AS011 (Abclonal) | IF (1:100) |
| Cy3 Goat Anti-Rabbit IgG (H+L) | AS007 (Abclonal) | IF (1:100) |
