# Supplementary material for: PIM2‐mediated phosphorylation contributes to granulosa cell survival via resisting apoptosis during folliculogenesis
Source: Clin Transl Med. 2021 Mar 9;11(3):e359. doi: 10.1002/ctm2.359 (PMC7943893; doi:10.1002/ctm2.359)
Supplement: Supplementary file 2 — Supporting information [file CTM2-11-e359-s004.pdf]

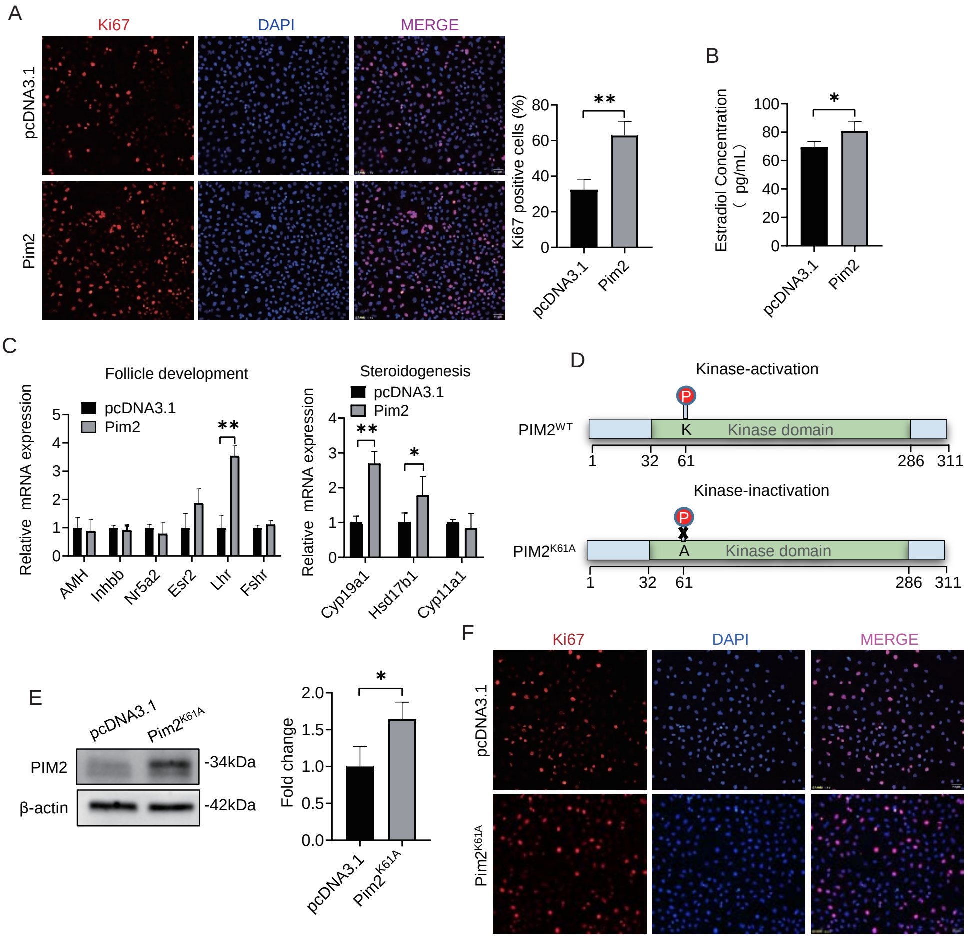

Figure S1. The pro-survival effect of PIM2 relies on its kinase activity.

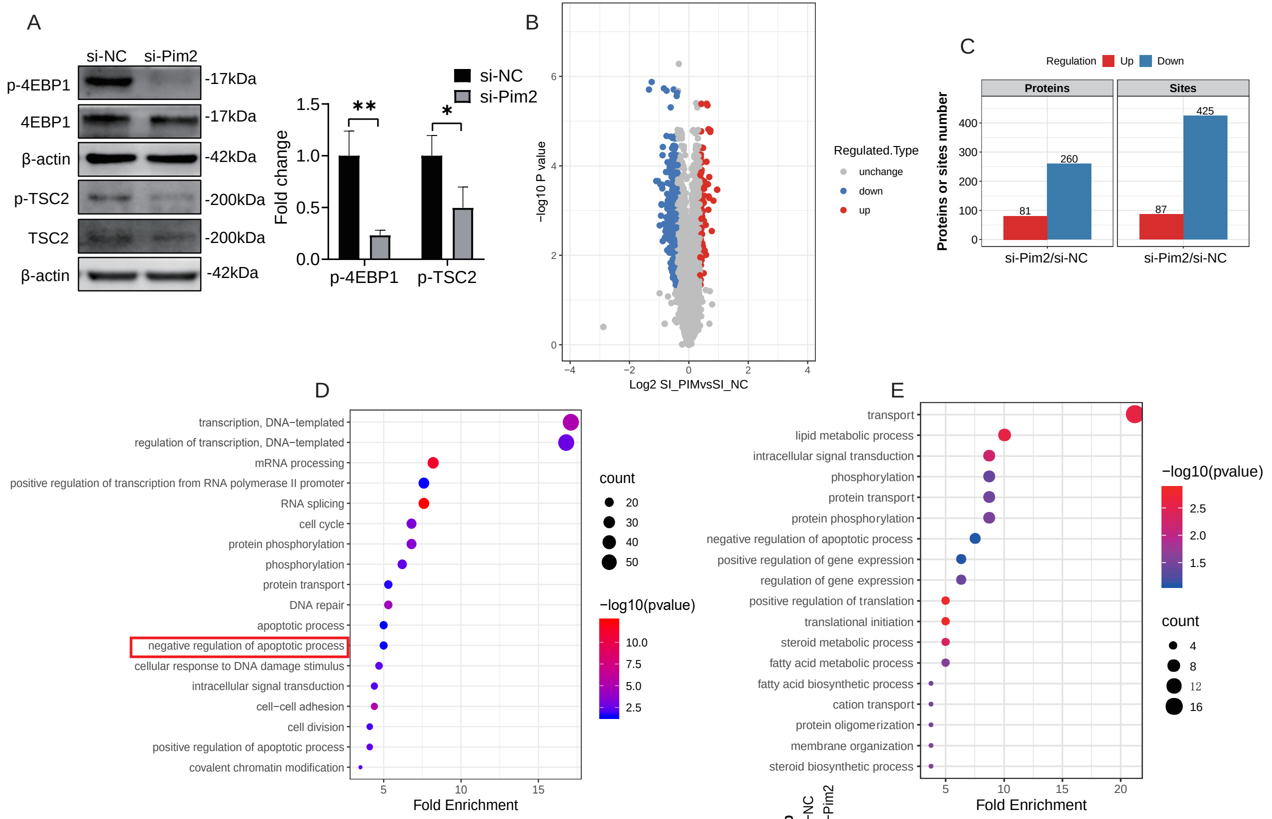

F

Gene name	Protein name	Protein accession	Phosphosite	Phosphopeptide
Dapk3	Death-associated protein kinase 3	O54784	S306	SHSSMPR
Raly	RNA-binding protein Raly	Q64012	T304	EHSQDTDAED
Lmnb1	Lamin-B1	P14733	T21	SAPATPLSPT

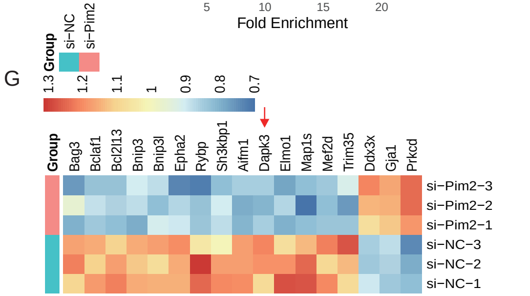

Figure S2. Phosphoproteomic screening to identify downstream phosphorylation events and PIM2 substrates.

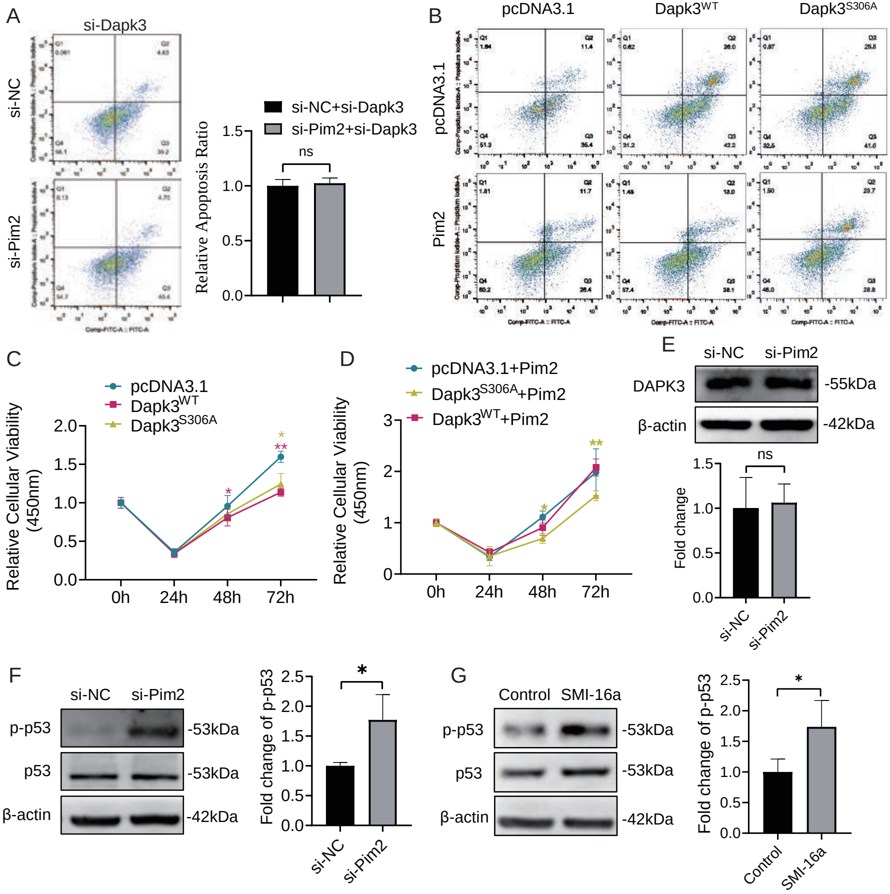

Figure S3. PIM2 kinase phosphorylates DAPK3 to resist apoptosis in GCs.

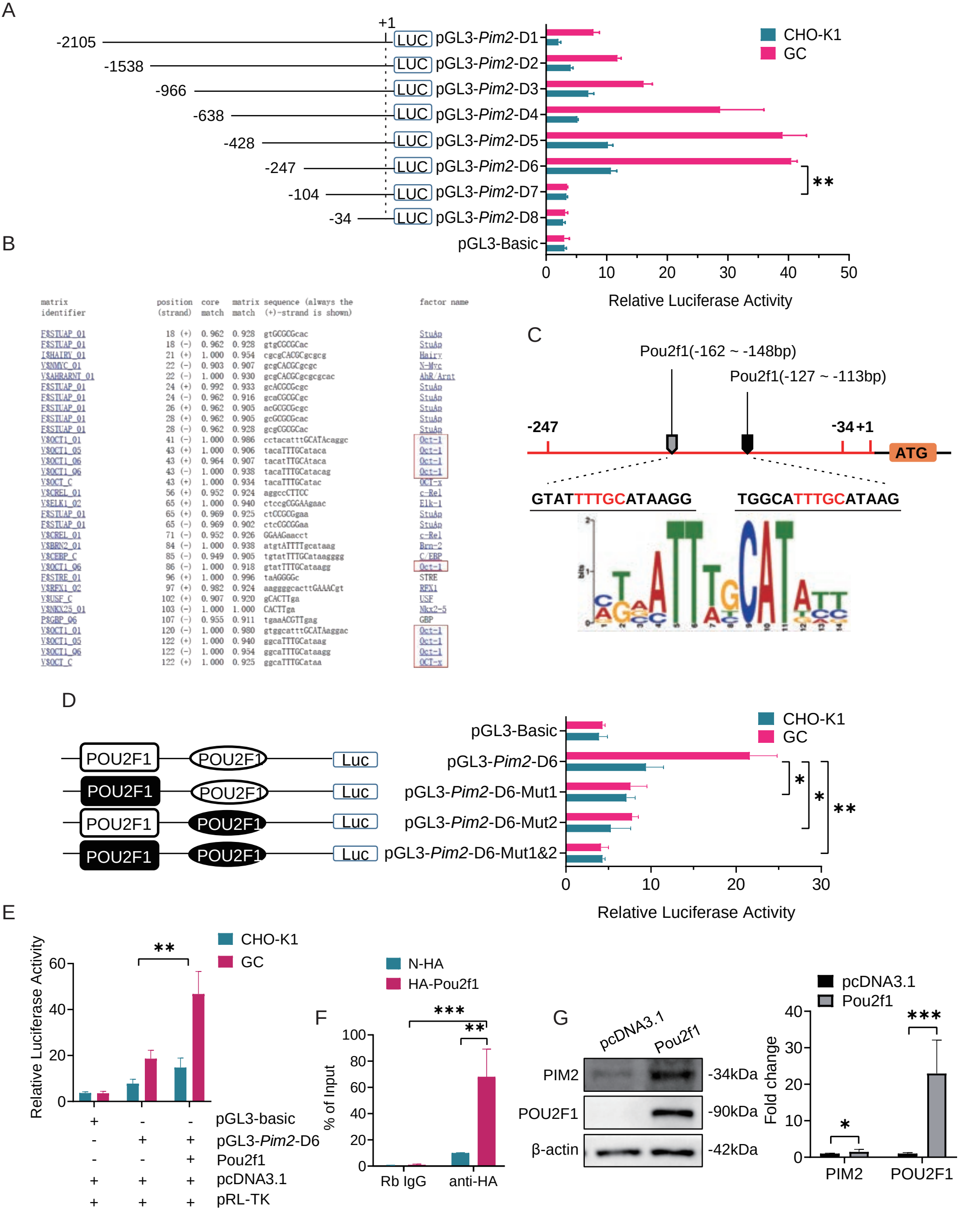

Figure S4. POU2F1 upregulates Pim2 promoter activity.

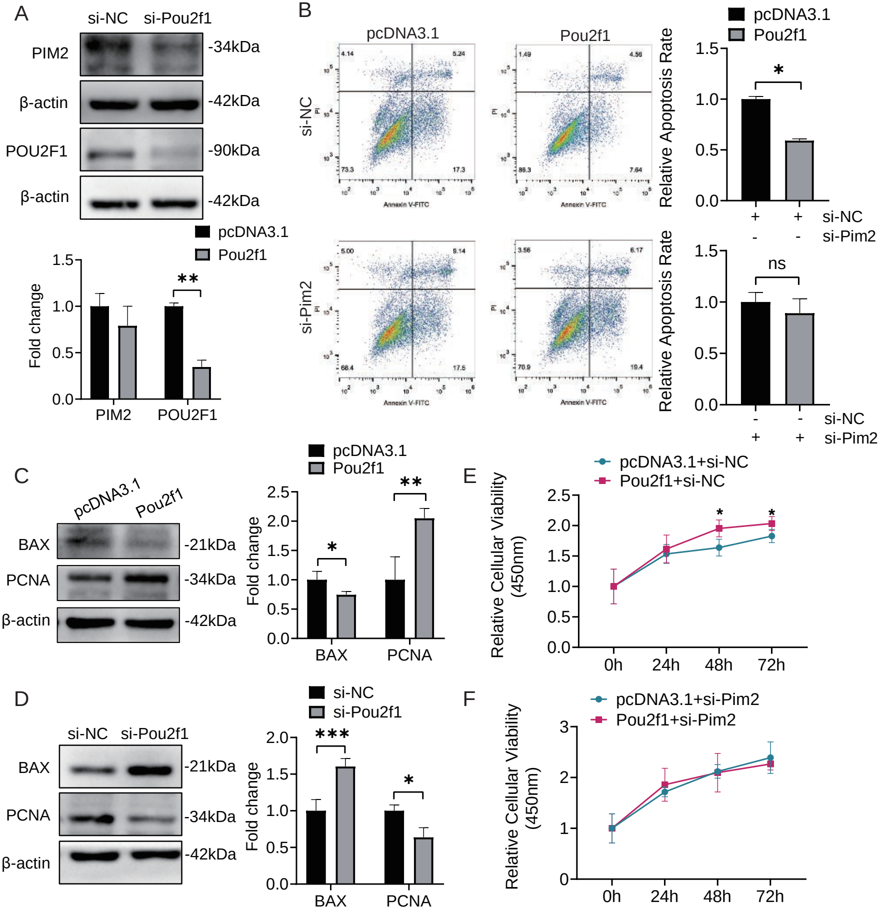

Figure S5. POU2F1 promotes GC survival in a PIM2-dependent way.

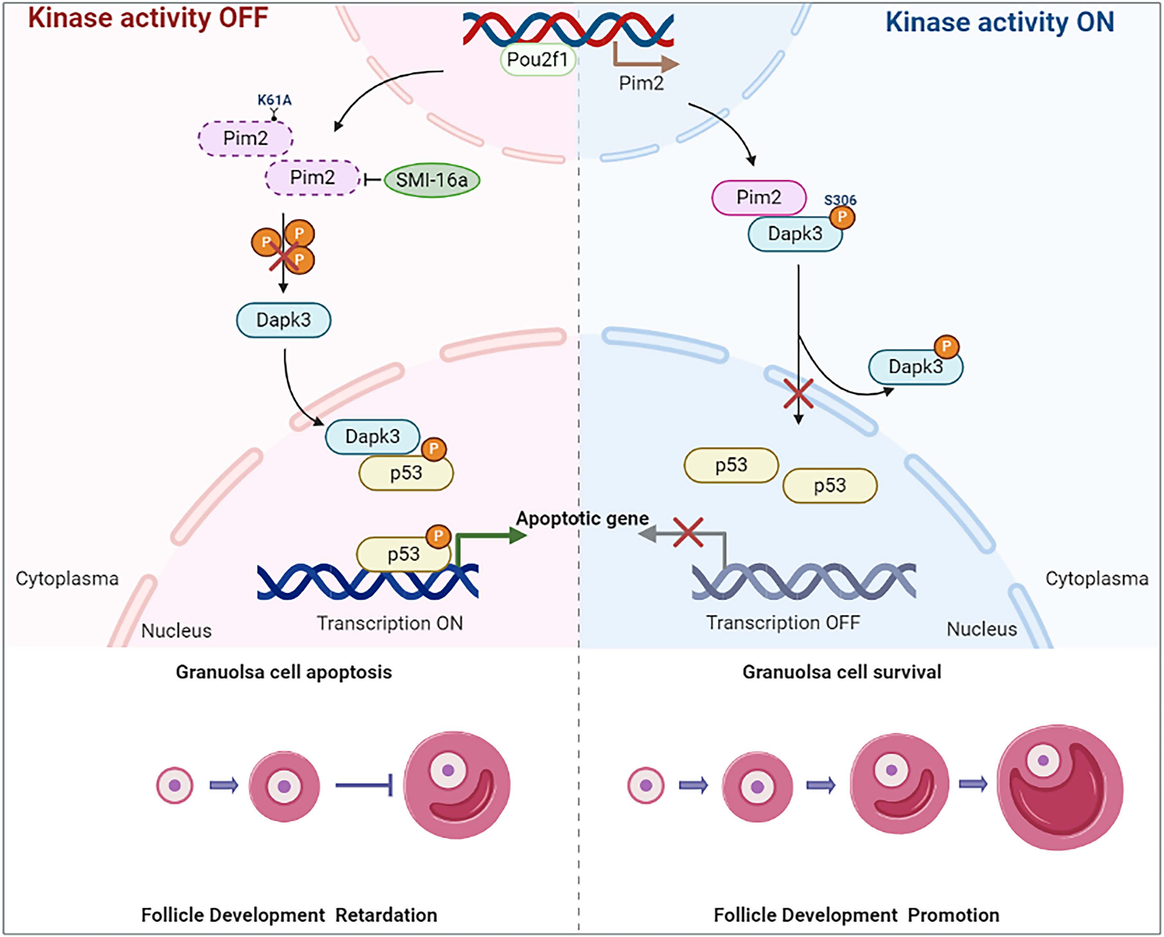

Figure S6. Schematic summary of the critical role of PIM2 kinase in GC survival and follicle development.
